# Assessment of meibomian glands using a custom–made meibographer in dry eye patients in Ghana

**DOI:** 10.1186/s12886-018-0869-0

**Published:** 2018-08-16

**Authors:** Eugene Appenteng Osae, Reynolds Kwame Ablorddepey, Jens Horstmann, David Ben Kumah, Philipp Steven

**Affiliations:** 1 Department of Optometry and Visual Science, Kwame Nkrumah University of Science and Technology, PMB, Kumasi, Ghana; 2 Department of Ophthalmology, Faculty of Medicine, University of Cologne, Cologne, Germany; 3 Cluster of Excellence: Cellular Stress Response in Aging – associated Diseases (CECAD), University of Cologne, Cologne, Germany

**Keywords:** Dry eye disease, Meibomian gland dysfunction, Ocular surface, Meibographer, Tear film

## Abstract

**Background:**

Meibomian Gland Dysfunction (MGD) is a leading cause of evaporative Dry Eye Disease (DED). This makes non-invasive meibography an important procedure in the clinical evaluation of DED patients. Our purpose was to conduct a lead-off investigation focused on the practicality of performing meibography in a developing country, with limited access to complex ophthalmic imaging systems, using a custom meibographer, as a step to future comparative studies on meibomian glands and DED in Africa.

**Methods:**

Meibomian glands(MG) in 76 upper eyelids (UL) and 49 lower eyelids (LL) in 1 eye each of 125 patients randomly selected from a patient population presenting with subjective DED symptoms at a clinic were photographed using a custom meibographer. Single frames were captured, and the MG area determined by intensity threshold segmentation and area calculation using Image J software. MG loss (MGL) was quantified by outlining its area and expressing it as a percentage of the total MG per Pult’s grading scheme. Dry eye measures included Tear Film Break Up - Time (TUBT), Schirmer’s test and Ocular Surface Staining (OSS). Symptoms were evaluated using the SPEED II questionnaire. Correlations between MGL and age, ocular signs and symptoms were analyzed by Pearson’s. Differences between comparable groups were analyzed by Mann - Whitney test; *p* < 0.05 was considered significant.

**Results:**

Overall mean MGL was 32.10% ± 25.0% (26.25% ± 22.40% for UL and 40.33% ± 26.70% for LL). MGL correlated significantly with age [*r* = 0.91, *p* = 0.001], SPEED scores [*r* = 0.90, *p* = 0.001], OSS [*r* = 0.75, *p* = 0.001] and TBUT [*r* = − 0.81, *p* = 0.001]. MGL scores were significantly higher in the UL than LL [U = 1293.5 *p* = 0.004].

**Conclusion:**

This study for the first time presents data on the status of Meibomian glands in Africa. It furthermore suggests that it is feasible to examine Meibomian glands using a custom meibographer in developing countries with limited access to complex imaging systems. It also demonstrates the benefit and cost-effectiveness of a simple device by the observed significant relations between meibomian gland loss and DED in these patients**.**

## Background

Dry eye disease (DED), synonymous to dysfunctional tear syndrome (DTS) or dysfunction of the lacrimal functional unit (LFU) is a multifactorial disease of the tears and ocular surface which results in symptoms of discomfort, visual disturbance and tear film instability with potential damage to the ocular surface [1, 2]. DED can broadly be classified as evaporative and aqueous – deficient DED or overlapping forms. In evaporative DED, there is decreased stability of the tear film due to abnormality in its lipid component. Aqueous – deficient DED on the other is linked to a reduced volume of the aqueous component of the tear film [3, 4].

The tear film lipids are produced by modified sebaceous glands in the eye lids, located within the tarsal plates [5]. These glands are called Meibomian glands (MG). Their secretion, also called meibum, is produced within these glands. It is secreted via ductal systems which open at the lid margin where it forms a thin layer on top of the aqueous and mucin tear components on the surface of the eye, preventing fast evaporation of tears and enhancing the lubricant properties of tears [5, 6]. A dysfunction of these glands is clinically referred to as Meibomian gland dysfunction (MGD). In MGD, there is poor quality and/or reduced volume of meibum – it is these alterations which lead to evaporative DED [7]. Different forms of MGD are distinguished as recently defined in the International Workshop on MGD Report. In particular low delivery and high delivery forms are distinguished that are related to dermatological diseases such as rosacea, systemic cicatrizing diseases such as pemphigoid or induced by drugs such as retinoids [2] .

Comparable to most developed countries, DED is a growing significant clinical problem in developing countries and emerging economies. Early studies conducted in these regions of the world have reported associations between DED and infectious disease like Trachoma and forms of malnutrition like vitamin A deficiency [8–13]. However, the current patterns of industrialization, modernization, urbanization and general socioeconomic transformations – including significant successes achieved at combating these infectious diseases and malnutrition in these areas could mean a present shift to other forms and causes of DED [14–22], such as MGD induced by aging, androgen deficiency, skin diseases, contact lens wear, etc.

It is widely known that MGD causes DED by altering the tear film lipids, destabilizing the entire tear film structure by disrupting the cohesivity between the various components. This causes tears to evaporate from the eye leading to reduced lubricity of the ocular surface therefore dryness at the ocular surface. Thus, assessment of the MG [5] is a crucial component in the comprehensive clinical evaluation and successful management of DED patients. The International Workshop on MGD suggests assessing for MGD in people by evaluating eyelid morphology, MG mass, MG expressibility, tear film lipid layer and MG drop-out or loss by meibography [7, 22].

Meibography is an imaging technique that provides an in - vivo means to assess the structure of the MG and makes it possible to view and quantify loss of glandular tissue (MGL) using a device called meibographer [24]. This is achievable because the technique allows for photographic documentation of Meibomian glands under specialized illumination situations. Meibography has undergone remarkable evolution but presently exists in two main forms – transillumination of the everted eye lids and direct illumination of the everted eyelids [24, 25]. The latter form is also called the non-contact (NCT) meibography, which has previously been described in different reports [23, 24].

NCT meibography comprises a slit lamp equipped with an infrared – charge coupled device with a video camera and infrared transmitting filter which makes it possible to view the glands in the everted lid non-invasively - without touching the eyelids. NCT meibography is believed to be a comfortable procedure for most patients compared to the transillumination method [25] but commercially existing NCT meibographers are expensive and thus may not be readily obtainable by most clinics especially those in developing countries.

Herein, we investigated the feasibility of conducting meibography in a developing country using a custom-made meibographer.

## Methods

### Construction of the customized meibographer

**Fig. 1:**
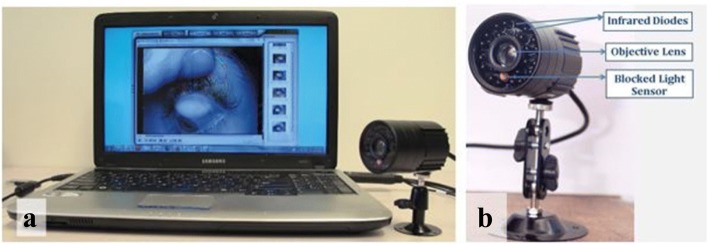
**a** Computer - meibographer set up. **b** Picture of meibographer with major components labelled

Following Pult’s suggestions [26], We obtained a simple infra – red video camera (Sunluxy SL – C221, Shen Zhen, China) and adapted it for near field imaging by an additional + 20 dioptre lens (Fig. 1). In addition, we blocked the light sensor of this camera to permanently cause it to image in dark or low levels of illumination – a condition which allows for the maximal illumination of the lids by the light source from the infrared diodes. The video camera is connected to a computer via a video –to – high speed serial bus converter / Logilink®VGA0001A (2direct GmbH, Schalksmühle, Germany).

### Clinical measurements and subject inclusion

Meibomian glands (MG) in one eye (overall 76 UL and 49 LL) of 125 patients, randomly selected from a patient pool presenting with subjective dry eye symptoms at a private practice in Kumasi Ghana, were photographed with the custom-made meibographer. Single frames were captured, and the MG area was determined. Additionally, a thorough slit lamp examination was conducted, and 2% topical fluorescein was applied to the ocular surface to measure Tear Film Break – Up Time (TBUT). We also graded Ocular Surface Staining (OSS) in 95 subjects using the Oxford Grading Scheme described by Bron et al. [27] using a Wratten # 12 filter (Kodak, New Jersey, USA). Symptoms of DED were evaluated using the SPEED II Questionnaire.

None of subjects included in the study had a history of ocular surgery including lid correction, neither did any report use of systemic drugs like isotretinoin [28] that could impair MG function. Further, none of the subjects had ever received treatment for MGD prior to the study.

### Meibomian gland evaluation

**Fig. 2:**
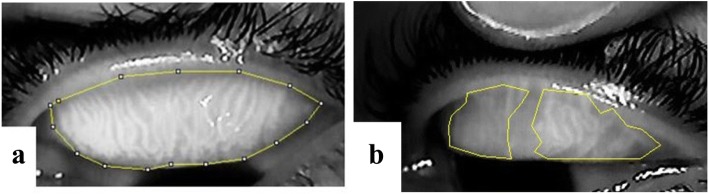
Meibographs of (**a)** an upper eyelid with ≈ 0%MGL (grade 0) (**b)** an upper eyelid with ≈50% MGL (grade 2)

Meibography images were evaluated by intensity threshold segmentation and area calculation with Image J software [29]. In detail, Meibomian Gland Loss (MGL) was determined by outlining the meibomian gland area present and expressing the area as a percentage of the total tarsal area as described by Pult’s et al. [26]. MGL was assigned grade 0 when there was approximately no (0%) glandular loss. Grades 1, 2, 3 and 4 represented ≤ 25%, 26–50%, 51–75% and > 75% of glandular loss (MGL) respectively. Representative images are shown in Fig. 2.

### Statistical analysis

Data was analyzed using SPSS version 20 (SPSS Inc., Chicago, USA). Normality was assessed using a graphical approach (visual inspection) method and the appropriate statistical testing performed subsequently. Relationship between MGL, age and ocular signs and symptoms were evaluated by Pearson’s Correlation. The differences between comparable categorical groups were investigated by performing the Mann – Whitney U test. Chi-square test was performed on certain distributions. *p* < 0.05 was considered significant.

### Ethical considerations

We adhered to the Declaration of Helsinki. Ethical approval was granted by the Committee on Human Research, Publication and Ethics at the School of Medical Sciences, Kwame Nkrumah University of Science and Technology/ Komfo Anokye Teaching Hospital, Ghana (CHRPE/ AP/448/16).

## Results

The summary of results of the major parameters of interests are represented in Table 1.

**Table 1:** Overview of results

Parameter	Mean ± SD	N	Range
Age [yrs]	46.20 ± 17.42	125	18.0–80.0
MGL [%]	32.10 ± 25.0	125	0.0–100.0
SPEED Scores	6.7 ± 3.9	125	2.0–6.0
TBUT Scores [sec]	6.2 ± 3.5	125	0.0–13.0
Oxford Grade of OSS	0.65 ± 0.75	95	0.0–2.0

*SD* Standard Deviation, *n* Number of patients/eyes, *MGL* Meibomian Gland Loss, *TBUT* Tear Break – Up Time, *OSS* Ocular Surface Staining

### Meibomian gland loss

**Fig. 3:**
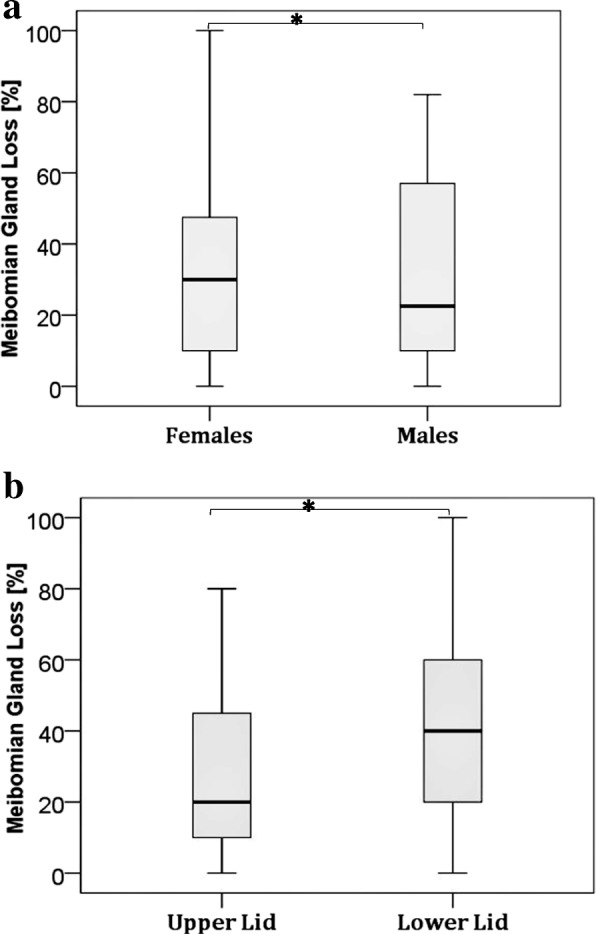
**a** Difference in MGL between gender groups and **b** Difference in MGL between upper and lower lids. * *p* < 0.05

As many as 84% of the patients studied showed some degree of Meibomian gland loss. The distribution of MGL based on Pult’s grading scheme is cross-tabulated in Table 2 for gender groups and eyelids. A Chi test revealed a statistically meaningful difference in the distribution of MGL between eyelid types (UL and LL) and between genders, **χ2**(12, *N* = 125) =28.49, *p* = 0.0047 We observed a significant difference in MGL between the UL (mean MGL = 26.25% ± 22.40) MGL and LL (mean MGL = 40.33% ± 26.70); [*U* = 1293.5, p = 0.004] but no significant difference between males (mean MGL = 32.56% ± 26.50) and females (mean MGL = 30.98% ± 23.70); [*U* = 1934. 5, *p* = 0.927] (Fig. 3a, b).

**Table 2:** Distribution of MGL grades between gender groups and eyelids

Pult’s Grades	Percentage distribution of MGL grades [%]			
	Upper eyelids [*n* = 76]	Lower Eyelids [*n* = 49]	Males [*n* = 62]	Females [*n* = 63]
Grade 0	19	10	14	16
Grade 1	40	27	42	28
Grade 2	25	24	16	34
Grade 3	13	32	22	21
Grade 4	3	7	6	1
Total %	100	100	100	100

### Tear film stability and ocular surface staining

There was a general reduced tear film stability among the study subjects. The overall mean TBUT was 6.5 s ± 3.5. Tear film stability scores among male patients were similar to that among females. Mean TBUT among males was 6.4 s ± 3.6 and that of females was 6.03 s ± 3.6; (*U* = 1813, *p* = 0.488).

The mean OSS value (for 95 subjects) was 0.65 ± 0.79. The difference in OSS between gender groups was not significant. We detected similar grades of ocular surface staining across male versus female groups. For males, mean OSS was 0.71 ± 0.85 and for females, mean OSS was 0.59 ± 0.74; (*U* = 1190 *p* = 0.609).

### Dry eye symptoms

The severity and frequency of dry eye symptoms was measured with the SPEED II questionnaire. An item on the questionnaire is graded a 0–4 Likert-type scale; where 0 means no symptoms and 4 means intolerable symptom. The composite score on the SPEED questionnaire ranges from 0 to 28 where 0 means and 28 would very severe and frequent symptoms. Average SPEED scores were similar between gender groups; males (6.65 ± 3.74) and females (6.81 ± 4.05); (U = 1943.0, *p* = 0.960).

### Relationship meibomian gland loss and age, speed, TBUT and OSS scores

**Fig. 4:**
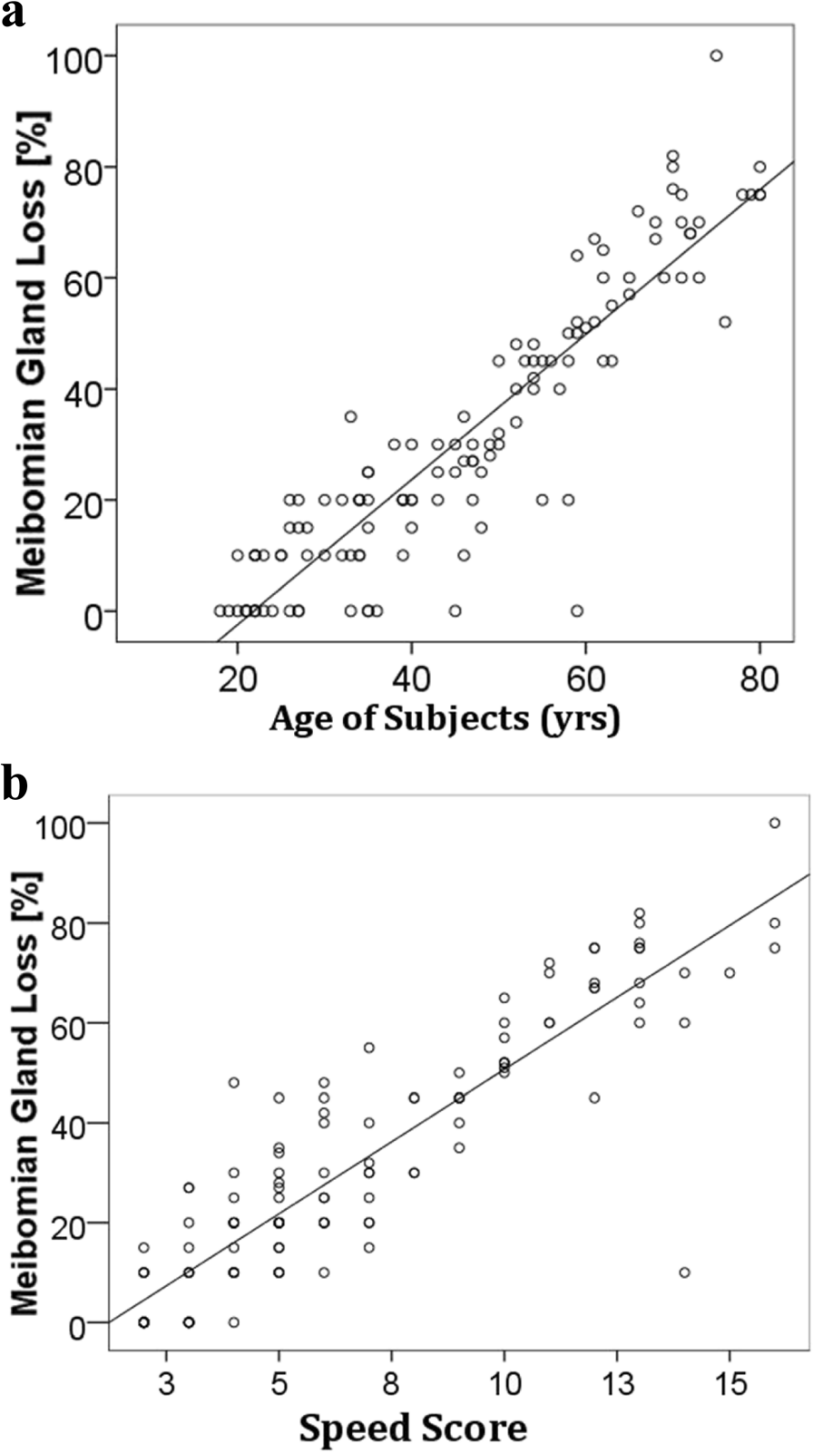
**a** Correlation between MGL and age and **b** Correlation between MGL and SPEED scores

**Fig. 5:**
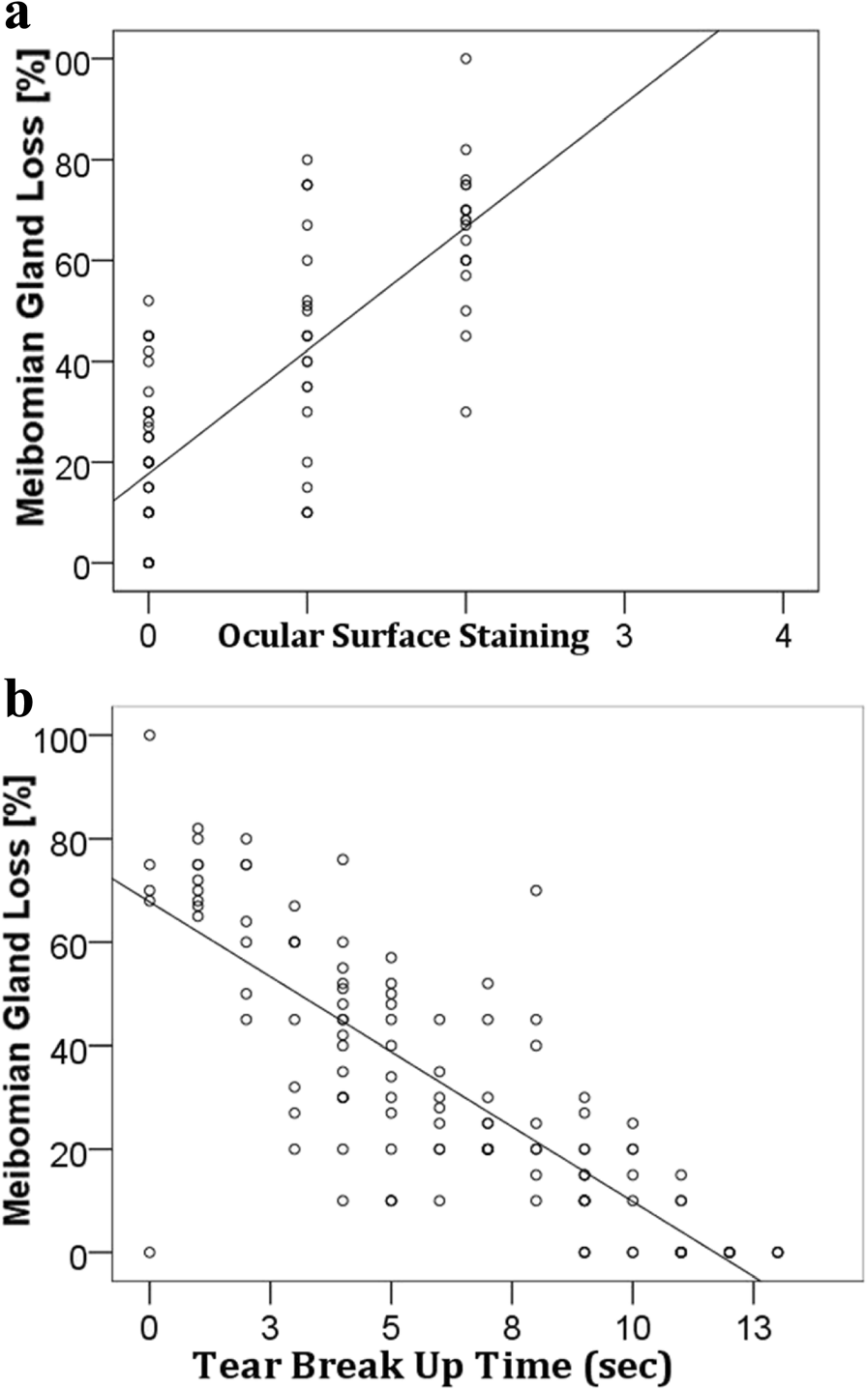
**a** Correlation between MGL and Ocular surface staining **b** Correlation between MGL and Tear break up time

We detected a strong positive correlation between MGL and age [*r* = 0.91, *p* = 0.001]. A similar correlation was also found between MGL and SPEED scores [*r* = 0.90, p = 0.001] and between MGL and Ocular Surface Staining [*r* = 0.75, p = 0.001]. There was a strong negative correlation between MGL and TBUT [*r* = − 0.81, p = 0.001]. (Fig. 4a,b) and (Fig. 5a,b).

## Discussion

Our findings from this first African study on meibomian glands and dry eye disease underscore Meibomian gland dysfunction as a major underlying cause of dry eye disease in this region of the world. Our findings hereby demonstrate a greater percentage (84%) of the total number of the subjects showed MGD of various degree or severity. While there is no reported absolute prevalence of MGD in Africa, our reported value is slightly higher than the 69.3% reported in the Beijing Eye Study [30] and 61.9% in the Japanese Studies [31]. It however, very high than 3.5% reported in the Salisbury Eye Evaluation Study [32] and 19.9% in the Melbourne Visual Impairment [33, 34].

The current understanding is that the prevalence of MGD is seemingly higher from studies conducted in Asian population compared to Caucasian populations [33]. As first-time study, our finding does not provide a complete picture of the prevalence of MGD among Africans; this warrants future studies necessary for knowing how MGD distributes on the Continent. It is important to state that these different studies report variable prevalence values due to differences in study design, especially variations in the diagnostic criterion for MGD. However, all of our study patients presented with subjective complaints of DED, suggesting a potential contribution of MGD to the dry eye disease process. Several different studies have also reported the presence of dysfunctional Meibomian glands in DED cohorts [35, 36].

It is understood that, MGD can lead to diffuse and specific changes in the gland itself resulting in reduced quality and volume of meibum [36]. MGD may present as hyposecretory action of the Meibomian gland, inspissation of meibum, change in colour and consistency of meibum, change in meibum oils composition and overall atrophy of the glands [4, 7, 36]. These changes prohibit the tear lipid layer from playing its normal role of preventing faster evaporation and enhancing the lubrication property of the tear film.

While the link between MGD and DED is well understood, the exact pathomechanism underlying MGD itself is poorly understood. Some experimental studies conducted to understand the disease process in MGD suggests the potential involvement of keratinization of the Meibomian gland ductal epithelium, chronic infection of the eyelids and inflammation [37, 38]. Others have also suggested the role of ageing, use of certain medications, dyslipidemia, and hyperosmolar stress at the ocular surface [39–42].

Additionally, it has been frequently reported that there is high prevalence of MGD among contact lens wearers [43, 44] even though the role of contact lens in the development of MGD is not completely understood yet. None of the subjects reported a history of contact lens wear but this does not preclude the likely role of other factors that could influence MGD-related DED. What draws our attention is that, infectious diseases (Trachoma) and malnutrition (vitamin A deficiency) have effectively being controlled (with some reports of successful eradication) in this region of Africa by global health interventional programmes like the World Health Organization’s SAFE strategy [**S** = surgery for trichiasis, **A** = Antibiotics **F** = Facial cleanliness, **E** = Environmental improvement] for trachoma control. Vitamin A supplementation programs have also seen significant success in these parts of the world [12–14, 18, 19]. We thus believe there are other risk factors and causes influencing DED - especially there may be greater role of environment and lifestyle in development of DED [13].

Higher temperatures and low humidity are believed to influence MGD and DED. Ghana like many other Africa countries have these characteristic climatic conditions and these may indirectly impact ocular surface health. Low humidity, dusty environment, high ventilation flow, prolonged use of computers and longer hours spent in air-conditioned environment among other factors can lead to ocular surface “injury” and inflammatory responses which mediate the vicious cycle of MGD and DED [33, 45].

In present day Ghana and many other developing nations, there is increased use of computers in schools and offices, many buildings and cars used in these hot climate regions are also air conditioned now. Much of the formerly agrarian settlements have also become heavily industrialized. This comes with alterations in the natural environment due increased construction of roads, factories, houses, and offices – all of which can cause concomitant increase in environmental pollution - directly or indirectly impacting health and disease including ocular surface health [13, 22, 45, 46].While this present study did not look at the potential contributions of these environmental factors to MGD and MGD-related dry eye, future studies should look at these factors may modulate MGD and MGD -related dry eye.

We found a significantly higher MGL in the UL than LL. This contradicts the findings of the Pult et al. who rather found greater MGL in the LL than UL [47]. While there is no definite explanation to this, earlier and current reports document a generally high number of glands in the UL than LL [5, 37]. In terms of morphology however, they report that LL MGs appeared more wider and shorter than UL MGs. We realize, this configuration may due to the relatively small physical space in the LL fornix; this perhaps makes it less easy to perform meibography on LL MGs than on UL MGs. We also think that; the upper eyelids do the greater part of movement during blinking and so its MGs are bound to experience mechanical (friction) forces that could affect its morphology and function and the overall development of MGD later in life – particularly in contact lens wearers [44, 48].

Further, we observed no significant difference in MGL between males and females. Our study is only a cross-sectional observational study with a limited number of participants. Therefore, the statistical interpretation of our findings should be considered in the same context. Several other studies suggest differences in phenotypes of MGD and therefore DED between male and female subjects [22, 33]. The function of the entire lacrimal functional unit, particularly the Meibomian glands is said to be regulated by sex - specific steroids [49–51] .

As has been frequently reported in other studies, we found a generally reduced tear film stability – measurable as low TBUT. A TBUT value less than the clinical average normal of 15 s is indicative of dry eye [52]. An overall of mean 6.2 ± 3.5 s was recorded in this study population, suggesting a general presence of DED population [53]. TBUT scores further correlated with meibomian gland dropout. This indicates the observed changes in the meibomian glands could be influencing the DED situation in our subjects. While correlations do not mean causations, several studies have reported that patients with meibomian gland dropout or MGD in general have issues with tear film instability. Patients with evaporative DED essentially have tears which dry away quickly from the ocular surface because they have poor quality and low volume meibum because of MGD [49, 54]. Dryness at the ocular surface can excite cascades of inflammatory response – including the expression inflammatory cytokines. Matrix metalloproteinase 9 (MMP9) is one of such expressed inflammatory factors – this is known to cause destruction of ocular surface integrity. MMP9s can slough off epithelial tight junctions of the conjunctival and cornea [55].

This causes irregularity in the cornea and conjunctiva. In the cornea, this can impair optical transmission of light resulting in blur or reduced visual acuity in most DED patients [56]. We detected such a change on slit lamp examination as ocular surface staining of various degrees following topical application of 2% fluorescein.

Dry eye patients may or may not be symptomatic. According to the SPEED scores we obtained, the subjects experienced one form or the other of DED -related symptoms. These may include pain, itchiness, redness, tearing, burning sensation, blur and itchiness [57, 58]. Different questionnaires for assessing dry eye symptoms demonstrate different degrees of sensitivity. The SPEED questionnaire was used in this population because it was simple to interpret, in the local Twi language, to some of patients when necessary. It also contained fewer items making it time - efficient to administer. Additionally, a previous study conducted in another region of Ghana concluded that SPEED questionnaire proves useful as valid measure of DED symptoms in the population [59]. We reported an average SPEED score of 6.7 ± 3.9 and a range of 2–6, comparable to the SPEED score of asymptomatic, mild, moderate and severe dry eye groups respectively reported in this other Ghanaian study [59]. Furthermore, it appeared useful but redundant to use a symptom assessment tool like the Ocular Surface Disease Index (OSDI) questionnaire because not many people in this Ghanaian community operated an ATM or drove a car [60]. This limitation presents an opportunity to design and validate region - specific questionnaire for evaluating DED patients in Ghana and other developing countries [13].

It is important to mention that, the clinical signs and symptoms of DED do not always correlate. Our findings however showed that MGL correlated well with age, DED symptoms (SPEED scores), tear film instability (TBUT) and ocular surface damage (OSS) among the subjects of this study. Atrophy of the Meibomian gland is known to increase with ageing. Plausible reasons offered to explain to this phenomenon include reduced cell cycling of MG acinar basal cells, reduced proliferative potential, hyperkeratinization and age-related co-morbidities [39, 40, 58]. Regarding relation between MGL, TBUT and OSS, there is a defined clear connection. When MGL occurs, volume and quality of meibum decreases, tear film evaporates faster from the ocular surface – inflammation may set in causing damage to ocular surface – which in turn causes and or exacerbates symptoms of dryness, pain and general ocular discomfort [57, 58].

## Conclusion

To summarize, this study for the first time presents data on the status of Meibomian glands in Africa. The observed results show that there is presently a potential link between DED and other factors (like MGD) other than infectious diseases and malnutrition in these regions. We have also demonstrated the feasibility of conducting meibography in DED patients using a custom-made meibographer in a developing country where there is limited access of complex imaging systems.

## Abbreviations

DED: Dry Eye Disease
LFU: Lacrimal Functional Unit
LL: Lower Lid
MG: Meibomian Gland
MGD: Meibomian Gland Dysfunction
MGL: Meibomian Gland Loss
NCT: Normal Contact
OSDI: Ocular Surface Disease Index
OSS: Ocular Surface Staining
SPEED: Standardized Patient Evaluation of Eye Dryness
TBUT: Tear Break Up Test
UL: Upper Lid
